# Functional responses of aquatic insect feeding groups in permanent semi-arid and arid wadis of Algeria

**DOI:** 10.3897/BDJ.14.e192613

**Published:** 2026-06-09

**Authors:** Kenza Meradi, Imene Benzina, Salah Meradi, Abdelkrim Si Bachir, Ahmed Yassine Kalbaza, Rabah Bounar

**Affiliations:** 1 Laboratory of Biodiversity and Biotechnological Techniques and Valorization of Plants Resources (BTB-VRV), Department of Natural and Life Sciences, Faculty of Sciences, Mohamed Boudiaf University, M'sila, Algeria Laboratory of Biodiversity and Biotechnological Techniques and Valorization of Plants Resources (BTB-VRV), Department of Natural and Life Sciences, Faculty of Sciences, Mohamed Boudiaf University M'sila Algeria; 2 Laboratory “Biodiversity, Biotechnology and Sustainable Development’’ LBBSD, Faculty of Nature and Life Sciences, University of Mostefa Benboulaid Batna 2, Batna, Algeria Laboratory “Biodiversity, Biotechnology and Sustainable Development’’ LBBSD, Faculty of Nature and Life Sciences, University of Mostefa Benboulaid Batna 2 Batna Algeria; 3 Laboratory of Environment, Health and Animal Production (LESPA), Department of Veterinary Sciences, Institute of Veterinary Sciences and Agronomic Sciences, University of Batna 1, Batna, Algeria Laboratory of Environment, Health and Animal Production (LESPA), Department of Veterinary Sciences, Institute of Veterinary Sciences and Agronomic Sciences, University of Batna 1 Batna Algeria

**Keywords:** functional feeding groups, forest microclimate, anthropogenic disturbance, environmental parameters, FFGs index, stream

## Abstract

This study reports the response of aquatic insect functional feeding groups (FFGs) to forest microclimatic conditions in a protected area and anthropogenic disturbance across four permanent wadis in the semi-arid and arid Aurès Region (north-eastern Algeria), comparing two wadis within a protected forested area with two exposed to domestic, agricultural, and industrial effluents. Aquatic insects and selected environmental parameters were sampled once per season. A total of 19,271 aquatic insects were collected, representing 58 taxa and 36 families distributed across seven orders. Across the recorded FFGs, the most abundant group was gathering collectors (67%), followed by filtering collectors (16.7%) and herbivore shredders (12.5%). Data were analysed using the Mann-Whitney U test, hierarchical clustering and canonical correspondence analysis. The FFGs indices were used as surrogates for stream ecosystem attributes to reveal functional changes of the studied wadis. The results indicate that the undisturbed wadis (Bouilef and El-Ma), located in a protected forest area under a semi-arid climate, showed higher functional diversity, characterised by the presence of all FFGs. The disturbed wadis (El-Hai and Gourzi), located in an arid region, display a low functional community, likely due to the degradation of water quality. This study showed that the forest microclimate, as well as the level of anthropogenic pressure, influenced the functional structure of the aquatic community. The autotrophy/heterotrophy index indicated that all studied wadis were heterotrophic. The shredder and filtering collector index were higher in forested wadis. The habitat stability index indicated that stable substrates were more abundant in undisturbed wadis. These results confirm the usefulness of the FFGs index as a relevant tool for understanding functional changes under a semi-arid to arid climate.

## Introduction

Freshwater ecosystems are dynamic systems whose structure and function are significantly influenced by hydrological, climatic and geomorphological circumstances at the watershed level (Machuca-Sepúlveda et al. 2024). In arid and semi-arid regions of North Africa, wadis are particularly sensitive to climatic variability and low and irregular precipitation (Gasith and Resh 1999, Arab et al. 2009, Benzina et al. 2021, Meradi et al. 2024). Even when streams remain permanent, their hydrological regimes are typically characterised by low flows and reduced faunistic diversity, which can strongly influence the composition and functional organisation of aquatic communities (Bonada and Resh 2013). However, permanent wadis embedded within forested microclimates, particularly those located in protected areas, may show different local conditions. Riparian forest cover provides shading that moderates water temperature, reduces evaporation and enhances habitat stability, while continuous inputs of coarse and fine particulate organic matter (CPOM and FPOM) from riparian vegetation sustain benthic food webs (Forio et al. 2020, Popescu et al. 2021). This forest microclimate allows permanent wadis to maintain a relatively high taxonomic and functional diversity of macroinvertebrate communities (Oester et al. 2025). Aquatic macroinvertebrates are widely recognised as reliable indicators of stream ecosystem condition, as their life cycles, physiological tolerances and feeding strategies respond sensitively to environmental changes (Kim et al. 2019, Gianopulos et al. 2021, Eh Rak and Samsudin 2025). Beyond taxonomic approaches (De Vries et al. 2021, Hu et al. 2022), functional classifications, based on FFGs, originally proposed by Cummins (1973) and later refined by Merritt et al. (2002), provide a mechanistic framework linking community structure to ecosystem processes. FFGs provide useful information about trophic organisation, organic matter dynamics and habitat functioning (Cummins 2021, Lima et al. 2022). The richness and abundance of FFGs have been successfully used to assess habitat condition and water quality across a wide range of aquatic ecosystems (El Yaagoubi et al. 2023, Cummins 2019). Numerous studies have demonstrated the sensitivity of FFGs to environmental parameters and anthropogenic disturbances in tropical, temperate and Mediterranean streams (Addo-Bediako 2021, Cortés‐Guzmán et al. 2022, Edegbene et al. 2023). However, functional assessments remain comparatively scarce in North Africa, particularly in arid and semi-arid contexts, where most studies have focused on taxonomic composition or abiotic factors. Only a few recent studies from Algeria and Morocco have applied FFGs or trait-based approaches to evaluate climate and anthropogenic impacts in semi-arid wadis (Meradi et al. 2024, El Yaagoubi et al. 2023, Nahli et al. 2022). In semi-arid regions, both climatic constraints and anthropogenic pressures, such as aridity, domestic, agricultural and industrial effluents, have profoundly altered water quality, substrate stability and organic matter composition (Benzina and Si Bachir 2018, El Yaagoubi et al. 2023, Guellaf et al. 2025). These disturbances typically favour pollution-tolerant and generalist taxa, particularly gathering collectors, while reducing the richness and abundance of sensitive functional groups, such as shredders, scrapers and filtering collectors (Bêche and Resh 2007). In disturbed wadis, anthropogenic pressures exert a greater influence on benthic communities than natural climatic factors. This influence primarily operates through the degradation of water quality, rather than through natural variations in flow or temperature associated with semi-arid climates (Hu et al. 2022, Machuca-Sepúlveda et al. 2024). Despite the FFGs ecological importance, functional assessments examining the influence of forest microclimatic conditions and anthropogenic disturbance in permanent wadis under semi-arid and arid climates remain very poorly documented. In this context, the present study provides one of the few functional evaluations of aquatic insect communities in permanent wadis of North Africa, highlighting how the forest microclimate of streams and human disturbances impact the community of FFGs. The main objectives of this study are the following: (i) to assess the influence of forest microclimate and anthropogenic disturbances on the abundance and richness of FFGs; and (ii) to apply the FFGs index as a proxy for stream ecosystem attributes to detect functional changes within the studied wadis under a semi-arid to arid context.

## Materials and methods

### Study region and wadis selection

**Figure 1.**
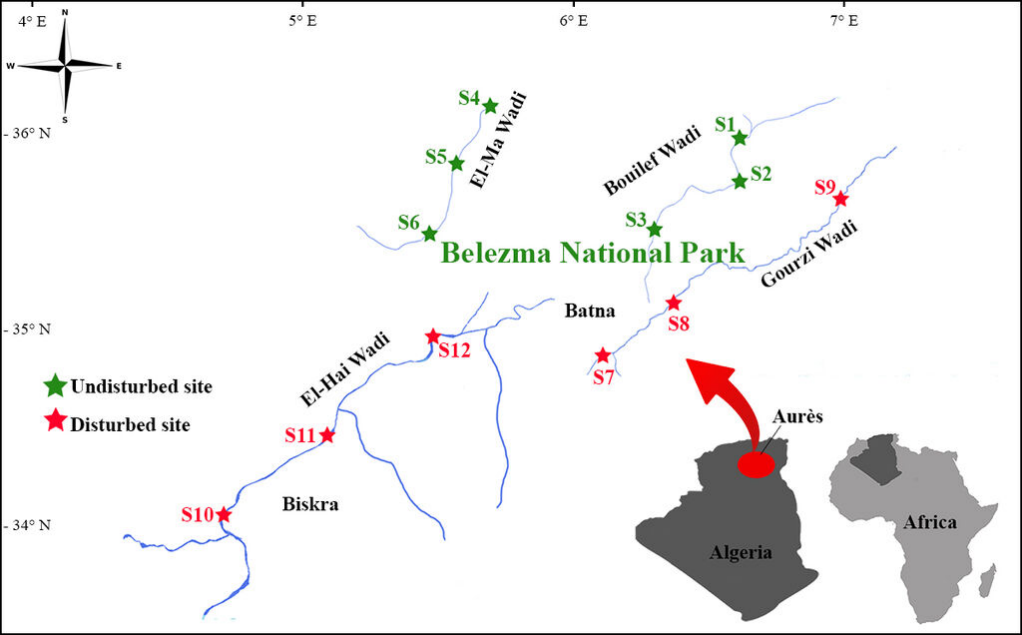
Location of study area and sampling sites. Bouilef wadi (S1, S2, and S3); El-Ma wadi (S4, S5, and S6); Gourzi wadi (S7, S8, and S9); El-Hai wadi (S10, S11, and S12). Green colour (undisturbed site); red colour (disturbed site).

This study was conducted in the Aurès Region, eastern Algeria, located in the eastern part of the Atlas Basin, between 34°30' and 36°05' N and between 5°45' and 7°20' E (Fig. 1). Two undisturbed wadis, El-Ma and Bouilef, are located within the Belezma Biosphere Reserve, near Batna City. The climate of the Reserve is generally semi-arid, characterised by cold winters and hot, dry summers at low altitudes (900–1200 m) (Boukerker and Si Bachir 2015). During the study period, total precipitation and mean air temperature were 366 mm and 14.4°C, respectively. The vegetation of the Reserve is dense and dominated by Atlas cedar (*Cedrus atlantica* (Endl.) Manetti ex Carrière), Aleppo pine (*Pinus halepensis* Mill.), holm oak (*Quercus ilex* L.), mastic tree (*Pistacia lentiscus* L.), blackberry (*Rubus ulmifolius* Schott) and various herbaceous species (Benchouala et al. 2023). In contrast, Wadi Gourzi is located outside the protected area and flows through Batna City, while Wadi El-Hai runs through the El-Kantara Region near Biskra City, which is characterised by an arid climate with warm winters. These two wadis, Gourzi and El-Hai, are considered disturbed and receive untreated domestic, agricultural and industrial effluents. These inputs result in high organic matter concentrations, nutrient enrichment and marked habitat degradation. Riparian vegetation along these disturbed wadis is sparse or severely degraded and channel morphology is frequently altered by sediment deposition and bank erosion (Meradi et al. 2024).

### Sampling design

Sampling was carried out from September 2021 to August 2022, covering four climatic seasons. In each wadi, three sampling sites were established, based on their location (upstream, middle and downstream), for a total of 12 sites. In the field, seven physical parameters of water were measured: pH, temperature (°C), total dissolved solids (TDS, mg l⁻¹) and electrical conductivity (EC, µS cm⁻¹), using a waterproof multi-parameter meter HI991300^®^ (HANNA Instruments); turbidity (NTU: Nephelometric Turbidity Units) with a turbidimeter (HACH Instruments); and water depth (cm) using a graduated wooden board. Additionally, water flow velocity was estimated using a flow scale: (1) very slow, (2) slow, (3) medium and (4) fast. In the laboratory, various chemical parameters were analysed, following standardised protocols for monitoring water quality (AFNOR Editions 2005). These parameters included salinity (mg l⁻¹), dissolved organic matter (DOM, mg l⁻¹), dissolved oxygen (DO, mg l⁻¹), nitrites (NO₂⁻, mg l⁻¹), nitrates (NO₃⁻, mg l⁻¹), ammonium (NH₄⁺, mg l⁻¹) and orthophosphates (PO₄³⁻, mg l⁻¹). At each site, aquatic insects were collected using a Surber sampler (25 × 25 cm frame, 500 µm mesh). All specimens were preserved in 4% formalin until sorting. In the laboratory, insects were counted and identified by genus or species using standard taxonomic keys (Tachet et al. 2002, Ochieng et al. 2019), then stored in 70% ethanol. Identified taxa were assigned to seven functional feeding groups following the classification of gathering collectors (GC), filtering collectors (FC), herbivore shredders (HSH), detrital shredders (DSH), scrapers (SC), algal piercers (APC) and predators (Pr). The various surrogate ratios of FFGs used to assess key stream ecosystem attributes, along with their proposed threshold values and ecological interpretations, are established according to Cummins (2019) and Cummins (2021) (Table 1).

### Data analysis

Total abundance corresponds to the total number of individuals, whereas relative abundance represents the proportion (or percentage) of a taxon relative to the total number of individuals. Richness "S" corresponds to the number of taxa in a given sample. The mean and standard deviation (mean ± SD) were calculated for each environmental variable in each of the studied wadis. To compare the relative abundance and richness of FFGs between disturbed and undisturbed wadis, we have used the Mann-Whitney U test with SPSS (2011). A hierarchical clustering analysis was performed using the UPGMA method and the Euclidean distance index, with Kovach (2007), to group sites according to their similarity in environmental parameters. The relationships between FFGs and environmental parameters were examined using Canonical Correspondence Analysis (CCA) with Hammer et al. (2001), which is based on correlations amongst variables across datasets.

## Results

### Environmental parameters

**Figure 2.**
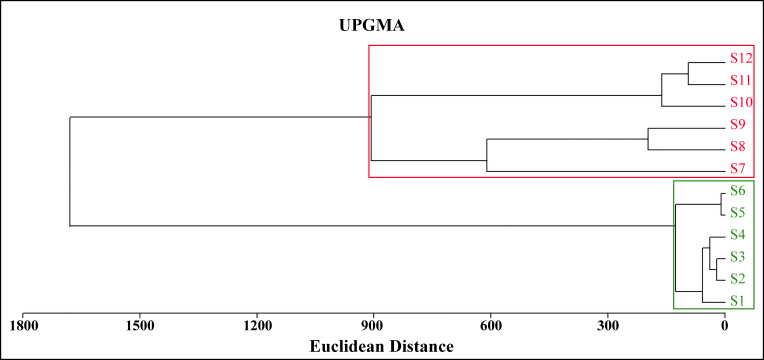
Dendrogram, based on Euclidean distance, calculated between sites according to their means of environmental parameters. UPGMA (Unweighted Pair Group Method with Arithmetic Mean). The red colour represents the disturbed sites and the green colour represents the undisturbed sites.

The analysis of environmental parameters revealed differences in water quality amongst the studied wadis. El-Hai and Gourzi Wadis showed high conductivity (2,250–2,440 µS cm⁻¹), elevated TDS, salinity, turbidity, DOM and nitrogen levels (NO₂⁻, NH₄⁺, PO₄³⁻), combined with very low dissolved oxygen (1.4 mg l⁻¹), largely exceeding standard limits. In contrast, Bouilef and El-Ma Wadis showed more stable conditions (Table 2). Hierarchical clustering analysis revealed two main groups amongst the studied sites. The first cluster includes sites of undisturbed wadis (Bouilef and El-Ma), indicating a high degree of similarity in their environmental parameters. The second cluster, distinct from the first, includes the sites of the disturbed wadis (El-Hai and Gourzi) (Fig. 2).

### Correlations between benthic insects FFGs and environmental parameters

**Figure 3.**
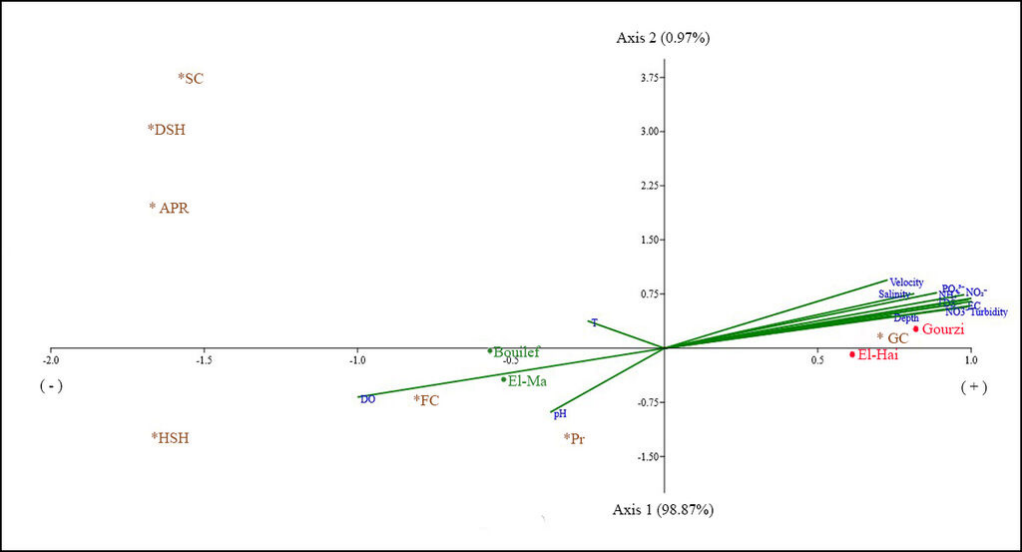
CCA between aquatic insect FFGs and environmental parameters. Axis 1 explained most of the inertia (98.87%), while Axis 2 explained only 0.97%. The abbreviations used for FFG groups are described in detail in the Materials and Methods section. Green colour Bouilef and El-Ma (undisturbed wadis). Red colour Gourzi and El-Hai (disturbed wadis).

The CCA performed between the FFGs and the environmental parameters reveals a strong correlation (Fig. 3). Axis 1 explained most of the inertia (98.87%) and showed a significant correlation between GC and the high values of various environmental parameters in Gourzi and El-Hai wadis (Fig. 3). The other functional groups (FC, HSH, APR, DSH and SC) are correlated with the high dissolved oxygen (DO) value in El-Ma and Bouilef wadis. Predators occupy a more central position in the ordination space, suggesting a low association with high values of environmental parameters. In addition, pH and temperature showed no correlation with the FFGs (Fig. 3, Suppl. material 1).

### Variation in the abundance and richness of FFGs across wadi disturbance levels

A total of 19,271 aquatic insects, belonging to seven orders, 36 families and 58 distinct taxa, were collected. The detailed list of taxa of the species recorded is presented in Table 3. Diptera was the most abundant order, representing 58.78% of the total individuals, followed by Trichoptera and Ephemeroptera, which accounted for 21.80% and 15.40%, respectively, with negligible contribution (from other groups?), followed by Coleoptera, Hemiptera, Odonata and Plecoptera orders (Table 3). According to FFGs, GC were the most abundant, with 12,912 individuals, representing 67.00% of the total insect abundance, followed by FC and HSH, with 3231 (16.76%) and 2426 (12.58%) individuals, respectively (Table 4). The presence of other groups was neglected (Table 4). With a recorded high relative abundance of GC (38%), FC (29.31%) and HSH (26.09%), W. Bouilef stands out amongst the studied wadis. Richness is also high in W. Bouilef, totalling 44 taxa evenly spread across different FFGs. Wadi El-Ma has a relative abundance of 43.15% for GC, followed by FC (38.59%), Pr (5.03%) and SC (1.42%). Wadi El-Hai is characterised by a high relative abundance of GC (98.57%) and a low abundance of FC (1.23%) (Table 4). The other FFGs are either absent or nearly so, which is reflected in low richness (only 10 taxa). Wadi Gourzi showed a relative abundance of GC and FC (58.89% and 7.69%, respectively). Richness is extremely reduced (7 taxa) and several FFGs are completely absent (Table 4). The Mann-Whitney test showed that undisturbed wadis (Bouilef and El-Ma) had significantly higher average abundances for SC (*p* = 0.005), APR (*p* = 0.000), DSH (*p* = 0.020), HSH (*p* = 0.000) and FC (*p* = 0.018) compared to disturbed wadis. No significant differences were found for GC (*p* = 0.527) and Pr (*p* = 0.103). Furthermore, the undisturbed wadis had significantly higher average richness than disturbed ones across all functional feeding groups (Table 5).

### Evaluation of FFGs' ratios

Analysing different FFG's ratios serves as a key indicator to detect functional changes within the studied wadis under a semi-arid to arid context (Table 6). The autotrophy/heterotrophy index (A/H) was below 0.75, which classifies all the studied wadis as heterotrophic. Wadis Bouilef and El-Ma both had SI values above the threshold of 0.25, while Wadis Gourzi and El-Hai had values below the threshold (SI = 0.00 and 0.002, respectively). The FCI was above the 0.5 threshold in Wadis Bouilef and El-Ma. Wadis Gourzi and El-Hai recorded null or very low values (FCI = 0.00 and 0.10, respectively). The HSI for wadis Bouilef and El-Ma were well above the 0.5 threshold; wadis Gourzi and El-Hai displayed low values (HSI = 0.13 and 0.10, respectively). Recorded values for PCI were neglected (Table 6).

## Discussion

### Functional structure of aquatic community 

The present study provides one of the few functional assessments of aquatic insect communities inhabiting permanent wadis in semi-arid and arid North Africa, highlighting how the forest microclimate and anthropogenic disturbances influence the FFGs community. Across all studied wadis, GC dominated benthic assemblages, comprising nearly two-thirds of total abundance. Baetidae and Caenidae mainly represented this FFG in undisturbed wadis and Chironomidae and Dixidae in disturbed wadis. GC are widely recognised as tolerant, plastic and generalists in their feeding strategy, consuming a wide range of food resources, particularly FPOM (Baker et al. 2023, Rimcheska et al. 2022). Consistent dominance of GC has been reported in semi-arid streams across Africa and other regions, where FPOM accumulation results from both natural organic enrichment and anthropogenic inputs (El Yaagoubi et al. 2023, Nahli et al. 2022, Ramos et al. 2025). FC, such as Hydropsychidae and Simuliidae, were the second most abundant FFG, particularly in the undisturbed wadis. They are typically associated with relatively stable flow conditions and sufficient current velocity to support effective suspension feeding (Fornaroli et al. 2019, El Yaagoubi et al. 2023). In contrast, the marked decline of FC in disturbed wadis suggests that water quality degradation, rather than hydrological constraints alone, limits their persistence. Elevated loads of wastewater-derived FPOM can clog or damage filtering structures, reducing feeding efficiency and survival (Ntloko et al. 2021).

Herbivore shredders were abundant only in the undisturbed wadis (Bouilef and EL-Ma), where riparian vegetation provides a continuous supply of food resources (Cummins et al. 1989). Within the framework of the River Continuum Concept (Vannote and Sweeney 1980), shredders play a key role in upstream reaches by converting CPOM into FPOM, thus supporting downstream collectors. Their absence in disturbed wadis is likely due to pollution-related changes in riparian vegetation, which reduce both food availability and suitable habitats for these sensitive taxa (Fu et al. 2015). Predators, such as Chaoboridae, Hydrophilidae, Veliidae and Dytiscidae, accounted for only a small fraction of total abundance, despite the high availability of potential prey, particularly GC. Scrapers, algal piercers and detrital shredders were recorded almost exclusively in forested wadis, indicating stable substrates, sufficient periphyton development and greater habitat heterogeneity (Cummins et al. 2005, Tomanova et al. 2006). According to Jun et al. (2011), the last FFGs, which are specialised in their diet, are sensitive to disturbances, explaining their low abundances in disturbed wadis. Similar results were reported by El Yaagoubi et al. (2023) and Henriques-Oliveira and Nessimian (2010), who found a lower relative availability of these FFGs at disturbed sites. The results of the Canonical Correspondence Analysis (CCA) confirm that the studied environmental parameters act as major predictors of changes in the functional structure of aquatic biota. In particular, GC shows a strong association with high values of environmental parameters (EC, TDS, turbidity, NO₂⁻, NO_3_⁻, NH₄⁺ and PO₄³⁻), confirming their tolerance to anthropogenic disturbances. In contrast, other specialised feeding groups, such as FC, HSH, APR, DSH and SC, require more favourable environmental conditions (El Yaagoubi et al. 2023, Peng et al. 2024). All these results strongly support our first hypothesis that forested microclimates and anthropogenic disturbance influence the functional structure of the aquatic community.

### FFGs index reveal changes in the ecosystem

According to Cummins (2019) and Cummins (2021), the FFGs index provided additional evidence of functional changes in the studied streams. The autotrophy/heterotrophy index indicated that all wadis were heterotrophic, but for contrasting reasons: riparian inputs in undisturbed wadis versus wastewater-derived organic matter in disturbed wadis. The same findings were reported by El Yaagoubi et al. (2023) in the Mediterranean wadis of the Moroccan Rif. Our results corroborate those reported by El Yaagoubi et al. (2023) and Peng et al. (2024), showing that the shredder index and filtering-collector index were high in undisturbed wadis, confirming the availability of CPOM and suspended FPOM from riparian vegetation. In disturbed wadis, these indices were negligible, reflecting the loss of these key functions. In addition, the habitat stability index (HSI) indicates that stable substrates are more abundant in undisturbed wadis (Bouilef and El-Ma), thus favouring the development of scrapers and filtering collectors. In contrast, disturbed wadis (Gourzi and El-Hai) are subject to constant and significant wastewater discharges, which reduce the number of stable substrates. This appears to result in poor attachment of scrapers and filtering collectors (Cummins 2019, El Yaagoubi et al. 2023).

## Conclusions

Our results show that permanent wadis located in forested areas under a semi-arid climate can support well-structured aquatic communities, including all the studied FFGs. In contrast, wadis affected by anthropogenic disturbances show reduced functional diversity. FFGs index appear to be a reliable indicator of river ecosystem attributes. Their assessment in the studied wadis provides us with a clear understanding of functional changes under a semi-arid to arid climate. Several previous studies conducted under similar conditions confirm our findings. Therefore, preserving these wadis is essential not only to maintain biodiversity and ecosystem functioning, but also to ensure the resilience of aquatic systems to increasing climatic and anthropogenic pressures.

## Supplementary Material

2B48E0E8-FFF8-5375-A979-3E5DA17B4C1E 10.3897/BDJ.14.e192613.suppl1 Supplementary material 1 CCA analysis results Data type Excel File: oo_1597820.xlsx https://binary.pensoft.net/file/1597820 Kenza Meradi

## Figures and Tables

**Table 1.** FFGs index surrogate stream ecosystem attributes, proposed ratio thresholds and interpretations (according to Cummins (2019) and Cummins (2021)). The abbreviations used for FFG groups are described in detail in the materials and methods section.

**Stream ecosystem attributes**	**FFGs index**	**FFGs ratios**	**Proposed thresholds and interpretations**
Autotrophic vs. Heterotrophic Energetics	Autotrophy/ Heterotrophy index (A/H)	SC + HSH + APC to DSH + GC + FC	**> 0.75**: Autotrophic stream dominated by Autochthonous DOM from algae and vascular plants); **< 0.75**: Heterotrophic stream that depends on Allochthonous DOM from riparian vegetation or wastewater).
CPOM vs. FPOM	Shredder index (SI)	DSH + HSH to GC + FC	**> 0.25**: Availability of CPOM from riparian vegetation for shredders.
Suspended vs. Storage FPOM	Filtering collector index (FCI)	FC to GC	**> 0.50**: Suspended FPOM from riparian vegetation; **< 0.50**: Stored FPOM from wastewater.
Stable vs. Unstable sediments	Habitat stability index (HSI)	FC + SC + HSH to DSH + GC	**> 0.50**: Stable substrates are more abundant than unstable substrates.
Top down vs. bottom up macroinvertebrate communities	Top-down predator index (PCI)	Pr to total FFGs	Predators > 20% either fast-reproducing predator species or presence of polyvoltine prey.

**Table 2.** Means and standard deviation (SD) of environmental parameters of sampled wadis: World Health Organisation (WHO). Bold characters indicate that the values did not follow the WHO standards (Cotruvo 2017).

**Parameters**	**Sampling Wadis**				**WHO Standards**
	**Bouilef**	**El-Ma**	**El-Hai**	**Gourzi**	
Depth (cm)	8.45 (± 4.48)	9.60 (± 5.61)	17.75 (± 4.27)	12.64 (± 4.39)	
T (^°^C)	27.52 (± 3.06)	20.37 (± 2.02)	23.98 (± 3.38)	21.81 (± 3.40)	
pH	7.97 (± 0.26)	**8.37 (**± **0.31)**	**8.24 (**± **0.29)**	7.74 (± 0.11)	6 to 8
EC (µS cm^−1^)	**772,58 (**± **23.91)**	**675.50 (**± **41.01)**	**2437.25 (**± **82.05)**	**2254.00 (**± **106.02)**	200 to 400
TDS (mg l^−1^)	586 (± 9.87)	539.50 (± 21.18)	**1228 (**± **38.86)**	**1051.83 (**± **32.39)**	500 to 1000
Salinity (%)	0.28 (± 0.07)	0.27 (± 0.05)	**0.86 (**± **0.10)**	**2.27 (**± **0.11)**	0.05 to 0.3
Turbidity (NTU)	13.98 (± 9.32)	20.52 (± 6.85)	**52.43 (**± **10.39)**	**52.75 (**± **3.05)**	5 to 30
DOM (mg l^−1^)	0.46 (± 0.24)	0.87 (± 0.66)	**5.73 (**± **0.23)**	**5.93 (**± **0.18)**	1 to 5
DO (mg l^−1^)	6.22 (± 0.12)	6.25 (± 0.20)	**1.42 (**± **0.18)**	**1.47 (**± **0.13)**	6 to 8
NO₂⁻ (mg l^−1^)	0.03 (± 0.01)	0.01	**1.16 (**± **0.45)**	**1.19 (**± **0.16)**	< 0.1
NO_3_⁻ (mg l^−1^)	1.36 (± 0.05)	1.61 (± 0.07)	2.31 (± 0.09)	2.45(± 0.14)	≤ 10
NH₄⁺ (mg l^−1^)	0.05 (± 0.04)	0.03 (± 0.02)	**1.06 (**± **0.85)**	**2.43 (**± **0.30)**	< 0.1
PO₄³⁻ (mg l^−1^)	0.00	0.03 (± 0.02)	**2.27 (**± **0.14)**	**3.31 (**± **2.12)**	< 0.05
Flow velocity	3.25 (± 0.43)	3.75 (± 0.00)	3.92 (± 0.29)	4.00 (± 0.00)	

**Table 3.** Taxon list of aquatic insects identified in the studied wadis, with their FFGs. und.: undetermined taxa (family or tribe). The abbreviations used for FFG groups are described in detail in the Materials and Methods section.

**Order / Family**	**Relative Abundance**	**Genera / Species**	**FFGs**
** Odonata **	0.1%		
Lestidae		*Lestes* sp.	Pr
** Ephemeroptera **	15.40%		
Baetidae		*Acentrella* sp. *Baetis* sp.	GC
Caenidae		*Caenis* sp.	GC
Leptophlebiidae		*Leptophlebia* sp. *Choroterpes* sp.	GC
** Plecoptera **	0.02%		
Capniidae		*Capnioneura* sp.	DSH
** Heteroptera **	0.7%		
Corixidae		*Corixa* sp. *Micronecta* sp.	APR
Hydrometridae		*Hydrometra* sp.	HSH
Veliidae		*Microvelia* sp.	Pr
Mesoveliidae		*Mesovelia* sp.	Pr
Notonectidae		*Notonecta glauca* (Linnaeus, 1758)	Pr
Nepidae		*Nepa* sp.	Pr
Gerridae		*Gerris* sp.	Pr
** Coleoptera **	3.2%		
Dytiscidae		*Yola* sp. *Platambus* sp. *Ilybius* sp. *Copelatus* sp. *Nebrioporus* sp. *Dytiscus* sp.	Pr
Elmidae		*Elmis* sp. *Limnius* sp.	SC GC
Haliplidae		*Haliplus* sp.	HSH
Hydrophilidae		*Crenitis* sp. *Hemisphaera* sp. *Berosus* sp. *Hydrophilus* sp.	Pr HSH
Scirtidae		*Hydrocyphon* sp. *Cyphon* sp.	APR
Staphylinidae		*Stenus* sp. *Stenus guttula* (Müller, 1821) Staphylinidae und.	Pr
** Trichoptera **	21.80%		
Brachycentridae		Brachycentrus sp.	FC
Hydropsychidae		*Hydropsyche* sp. *Cheumatopsyche* sp.	FC
Hydroptilidae		*Hydroptila* sp.	APR
Glossosomatidae		*Glossosoma* sp.	SC
Sericostomatidae		*Sericostoma* sp.	HSH
** Diptera **	58.78%		
Dixidae		*Dixa* sp. Dixidae und.	GC
Ceratopogonidae		Ceratopogonidae und.	Pr
Chaoboridae		*Chaoborus* sp.	Pr
Chironomidae		*Chironomus* sp.	GC
Psychodidae		*Psychoda* sp. *Clogmia* sp. Psychodidae und.	GC
Thaumaleidae		Thaumaleidae und.	SC
Syrphidae		*Eristalis* sp.	GC
Ptychopteridae		*Ptychoptera* sp.	GC
Stratiomyidae		Stratiomyidae und.	GC
Simuliidae		Simuliinae und. Prosimuliinae und.	FC
Tipulidae		*Tipula* sp.	DSH
Tabanidae		*Tabanus* sp.	Pr
Culicidae		*Culex* sp.	FC

**Table 4.** Total abundance and richness of FFGs in the studied wadis. The abbreviations used for FFG groups are described in detail in the Materials and Methods section.

	**FFGs**															
**Wadis**	**Pr**		**SC**		**GC**		**FC**		**HSH**		**DSH**		**APR**		**Total**	
	**Ab**	**S**	**Ab**	**S**	**Ab**	**S**	**Ab**	**S**	**Ab**	**S**	**Ab**	**S**	**Ab**	**S**	**Ab**	**S**
Bouilef	244	18	150	1	3301	11	2546	5	2266	3	13	1	165	5	8685	44
El-Ma	74	7	21	3	634	7	567	4	160	1	10	2	3	1	1469	25
El-Hai	13	4	4	1	8910	2	112	3	0	0	0	0	0	0	9039	10
Gourzi	4	1	1	1	67	4	6	1	0	0	0	0	0	0	78	7
**Total individuals**	335		176		12912		3231		2426		23		168		19271	

**Table 5.** Total abundance and richness of FFGs between undisturbed wadis (Bouilef and El-Ma) and disturbed wadis (Gourzi and El-Hai). Significant values are shown in bold. The abbreviations used for FFG groups are described in detail in the Materials and Methods section.

**FFGs**	Disturbance degree	**Total Abundance**	Mean rank	Sum of ranks	*p*-value	**Richness**	Mean rank	Sum of ranks	*p*-value
**SC**	undisturbed	171	29.15	699.50	**0.005**	3	29.19	700.50	**0.004**
	disturbed	5	19.85	476.50		2	19.81	475.50	
**APR**	undisturbed	168	30.00	720.00	**0.000**	5	30.00	720.00	**0.000**
	disturbed	0	19.00	456.00		0	19.00	456.00	
**DSH**	undisturbed	23	27.00	648.00	**0.020**	2	27.00	648.00	**0.019**
	disturbed	0	22.00	528.00		0	22.00	528.00	
**HSH**	undisturbed	2426	36.50	876.00	**0.000**	3	36.50	876.00	**0.000**
	disturbed	0	12.50	300.00		0	12.50	300.00	
**GC**	undisturbed	3935	23.23	557.50	0.527	12	31.81	763.50	**0.000**
	disturbed	8977	25.77	618.50		6	17.19	412.50	
**FC**	undisturbed	3113	29.21	701.00	**0.018**	5	31.25	750.00	**0.000**
	disturbed	118	19.79	475.00		3	17.75	426.00	
**Pr**	undisturbed	318	27.73	665.50	0.103	23	28.42	682.00	**0.045**
	disturbed	17	21.27	510.50		4	20.58	494.00	

**Table 6.** FFGs ratios calculated for the studied wadis. The abbreviations used for FFG index and ratios are described in detail in the Materials and Methods section.

		**Ratio values**			
**FFGs** **Index**	**FFGs Ratios**	**W. Gourzi**	**W. El- Hai**	**W. Bouilef**	**W. El-Ma**
A/H	SC + HSH + APR / DSH + GC + FC	0.13	0.00	0.36	0.45
SI	DSH + HSH / GC + FC	0.00	0.002	0.59	0.48
FCI	FC / GC	0.00	0.10	0.77	0.83
HSI	FC + SC + HSH / DSH + GC	0.13	0.10	1.10	1.56
PCI	Pr / Total FFGs	0.00	0.01	0.02	0.03
